# Characteristics of Lung Microbiota in Children's Refractory *Mycoplasma pneumoniae* Pneumonia Coinfected with *Human Adenovirus B*

**DOI:** 10.1155/2022/7065890

**Published:** 2022-01-17

**Authors:** Wenxiang Zhou, Jinglong Chen, Zhimin Xi, Yanyan Shi, Libo Wang, Aizhen Lu

**Affiliations:** ^1^Division of Internal Medicine, Children's Hospital of Xiamen, Xiamen, Fujian, China; ^2^Division of Pulmonary Medicine, Children's Hospital of Fudan University, Shanghai, China

## Abstract

**Background:**

Both *M. pneumoniae* and human adenovirus (HAdV) are common causative agents of lower respiratory tract infection in children; nonetheless, the lung microbiota in patients with coinfection of HAdV and *M. pneumoniae* remain unexplored.

**Methods:**

Thirty-two children, diagnosed with refractory *M. pneumoniae* pneumonia (RMPP), entered into the one-year study from July 1, 2019 to June 30, 2020. Among them, twenty-one entered into the *M. pneumoniae* monoinfection (MP) group and eleven entered into the *M. pneumoniae* and HAdV coinfection (MP&ADV) group. The characteristics of the clinical findings were examined, and the lung microbiota was analyzed by metagenomic next generation sequencing (mNGS).

**Results:**

Eleven patients in the MP&ADV group were coinfected with *human mastadenovirus species B*. The fever days lasted for significantly longer periods in the MP&ADV group than in the MP group (*P* < 0.05). The percentage of CD16^+^CD56^+^ cells was significantly higher in the MP&ADV group than that in the MP group (*P* < 0.05). There were no significant differences in *α*-diversity between the MP and MP&ADV groups, but the *β*-diversity was clearly higher in the MP&ADV group than that in the MP group (*P* < 0.05). At the microbial level, the top phylum of the MP BALF microbiota was *Tenericutes*; in contrast, it was *Preplasmiviricota* in the MP&ADV BALF. There were significant differences in the relative abundance of *Tenericutes* and *Preplasmiviricota* between the two groups (*P* < 0.001). There was a strong positive correlation between *human mastadenovirus B* and fever days, *M. pneumoniae* and level of IgA, and a strong negative correlation between *Mycoplasma pneumoniae* and PCT.

**Conclusions:**

In RMPP, the BALF microbiota in children with mono *M. pneumoniae* infection was simpler than those with coinfection with *human mastadenovirus B*. Prolonged fever days were associated with *human mastadenovirus B* coinfection.

## 1. Introduction

*M. pneumoniae* is an important pathogen of community-acquired pneumonia in children [1, 2]. Infection by *M. pneumoniae* is generally self-limited but can lead to severe pneumonia requiring intensive care [3]. It can also cause multiple extrapulmonary manifestations, involving the skin, musculoskeletal, nervous, hematological, digestive, and renal systems [4]. In addition, damage to the epithelial cells and cilia of the human airway can occur [5], affecting the function of the mucus-ciliary clearance system [5, 6] and host immunity [7], in turn increasing the rate of coinfection by opportunistic pathogens. The coinfection rate has been reported to reach 27%–48% in *M. pneumoniae* pneumonia (MPP) [8, 9] and are also closely associated with refractory MPP (RMPP) [10].

Human adenovirus (HAdV) is an important pathogen of the respiratory tract in children, accounting for 4–10% of pediatric community-acquired pneumonia [11]. *M. pneumoniae,* co-infected with HAdV in children, has been reported with more serious clinical manifestations [12]; however, the pathogenesis of coinfection of HAdV and *M. pneumoniae* has not been clarified. The microbiota has been reported to change during the lower respiratory tract (LRT) infection, and the change is closely related to the course or prognosis of pneumonia [13, 14]. Nevertheless, it is currently unknown whether the microbiota in the LRT is associated with coinfection of HAdV and *M. pneumoniae*. We hypothesized that knowledge of the characteristics of the pediatric LRT microbiota in RMPP, coinfected with HAdV, may offer opportunities to uncover the mechanisms of pathogenesis of the coinfection, which is an unmet clinical need. Here, a case-control study was designed to achieve this goal.

## 2. Methods

### 2.1. Subjects and Groups

This is a prospective cohort study, in which the RMPP cases, hospitalized in the Children's Hospital of Fudan University from July 1, 2019, to June 30, 2020, were screened. RMPP is referred as clinical manifestations and the pulmonary images of *M. pneumoniae* pneumonia, showing deterioration after regular macrolide antibiotics treatment for more than seven days [15]. If signs of bronchial obstruction exist such as atelectasis or consolidation, bronchoscopy was performed. All RMPP cases received bronchoscopy, and informed consent form for bronchoscopy was obtained from the guardians. *M. pneumoniae* infection was confirmed by serological tests, positive for *M. pneumoniae* IgM or an IgG antibody titer ≥1 : 160 or with a ≥4-fold increase (SeroMP^TM^ IgM and SeroMP^TM^ IgG test kit, Savyon Diagnostics Ltd) and by polymerase chain reaction showing >2,500 copies of *M. pneumoniae* genome per mL in the nasopharyngeal aspirate or BALF (*M. pneumoniae* nucleic acid amplification fluorescence detection kit, Daan Gene Co., Ltd., Guangzhou) [16]. HAdV infection was confirmed by a positive result of HAdV antigen from nasopharyngeal aspirates or BALF (D^3^ Ultra DFA Respiratory Virus Screen & ID kit, Diagnostic Hybrids, Inc). Patients with single *M. pneumonia* infection entered into the MP group, and those coinfected with *M. pneumoniae* and HAdV entered the MP&ADV group. The exclusion criteria were (i) detection of any other pathogens in the patients' blood, nasopharyngeal aspirate, sputum, or BALF via culture, viral antigen detection assays, or serum tests and (ii) patients with chronic diseases, immune deficiencies, heart diseases, or using immunosuppressive drugs. The ethical application of this study was approved by the ethics committee of Children's Hospital of Fudan University on March 29, 2016 (No. 2016-87).

### 2.2. Clinical Characteristics and Laboratory Findings

Clinical information was collected, which included age, gender, and hospitalization days. The laboratory findings before bronchoscopy were also recorded, which comprised white blood cell (WBC) count, lymphocyte (LY) count, lymphocyte percentage (LY%), neutrophil (Neu) count, neutrophil percentage (Neu%), C-reactive protein (CRP), creatine kinase isoenzyme-MB (CK-MB), lactate dehydrogenase (LDH), levels of procalcitonin (PCT), D-dimer, alanine aminotransferase (ALT), aspartate aminotransferase (AST), lymphocyte subpopulations including percentages of CD3^+^ T cells, CD3^+^CD4^+^T cells, CD3^+^CD8^+^T cells, CD16^+^CD56^+^ T cells, and CD19^+^ T cells, and lastly, humoral immunity that included immunoglobulin G (IgG), immunoglobulin M (IgM), immunoglobulin A (IgA), and immunoglobulin E (IgE).

### 2.3. BALF Specimen Collection

Bronchoscopy was performed under conscious intravenous sedation with midazolam. Topical anesthesia of the larynx, trachea, and carina was achieved with 2% lidocaine (Sanchine, China), the bronchoscope was wedged in the lesion's segment or lobe, and the lavage was performed with three aliquots of sterile saline (Baxter, China), 1 ml/kg each, with a suction pressure of 100 mm Hg. All BALF samples were then immediately processed and stored according to the requirements of the laboratory.

### 2.4. DNA Extraction and Metagenomic Sequencing

DNA extraction from the BALF was performed as described in [17]. Briefly, 1 ml BALF was digested with 50 *μ*l protease K at 60°C for 20 min and then placed at 4°C for 5 min. The sample was transferred into a sterile 5 ml tube, followed by brief centrifugation, and the DNA was extracted using the TIANamp Magnetic DNA kit (DP710-t2, Tiangen, China), according to the manufacturer's protocol. A no-template control (NTC) was performed for PCR. The quantity was assessed using the Qubit 2.0 fluorometer (Thermo Fisher Scientific, USA), and the quality of DNA was evaluated using the Nanodrop 8000 spectrophotometer (Thermo Fisher Scientific, USA). BALF DNA was fragmented into 150–300 bp size range by using the Bioruptor Pico Plus (Diagenode, Belgium) with the ultrasonication parameters as follows: 30 s on, 30 s off; 10 cycles. The DNA library was constructed using the KAPA HyperPrep kit (KAPA Biosystems, USA), according to the manufacturer's protocol. The library was qualified with Agilent 2100 (Agilent Technologies, CA) and sequenced on Illumina NextSeq 550Dx (Illumina, USA) using 75 bp single-ends.

### 2.5. Bioinformatics

Raw sequencing data were split by using bcl2fastq2, and clean reads were screened using Trimmomatic by removing low-quality reads, adapter contamination, duplications, and short (length < 35 bp) reads [17]. Bowtie2 was then used to align with the human genome, and the unaligned sequences were retained. Kraken2 was used to identify the species contained in the sample, and Bracken was used to predict the actual relative abundance of the species in the sample.

### 2.6. Statistical Analysis

The statistical analyses were performed using the SPSS software (IBM, version 25.0); *P* < 0.05 was defined as statistically significant. Other statistical analyses were performed using the R software (v4.0.1). Alpha diversity was measured using the Shannon index and Simpson index. Beta diversity was evaluated using the Bray–Curtis measure, compared by using the Wilcoxon rank sum test between the MP&ADV and the MP groups, and visualized with the principal coordinate analysis (PCoA) plot. The “vegan” R package was used to perform permutational multivariate analysis of variance (PERMANOVA) to analyze the Bray–Curtis distance in the MP&ADV and MP groups. The Kruskal–Wallis rank-sum test (R package “kruskal.test”) was used to test differential relative abundance of taxonomic groups at the genus level. The “cor.test” R package was used to assess Spearman's correlations between clinical characteristics and the relative abundances of the genera, and the FDR correction was used to adjust all *P* values.

## 3. Results

### 3.1. Clinical Characteristics

In this study, 21 subjects, consisting of 11 males and 10 females, were entered into the MP group, and 11 subjects, consisting of 7 males and 4 females, were entered into the MP&ADV group. All eleven patients in the MP&ADV group were coinfected with *human mastadenovirus species B*. There was no significant difference in gender distribution, age, or inpatient days between two groups (Table 1). The fever days were significantly longer in the MP&ADV group than those in the MP group (*P* < 0.05) (Table 1). There were 13 patients (61.9%) with extrapulmonary manifestation in the MP group, including 8 with myocardial damage, 4 patients with liver damage, and 1 with liver damage and urticaria. There were 5 patients (45.5%) with extrapulmonary manifestation in the MP&ADV group, including 4 with myocardial damage and 1 with liver damage. There was no significant difference in the extrapulmonary manifestation or in treatments with methylprednisolone or IVIG between the two groups (*P* > 0.05, respectively) (Table 1).

### 3.2. Laboratory Findings

In laboratory findings, there were no significant differences in the WBC count or levels of CRP, CK-MB, PCT, LDH, D-dimer, ALT, or AST between 2 groups (*P* > 0.05) (Table 2). In cellular immunity, there were also no significant differences in the percentages of CD3^+^, CD3^+^CD4^+^, CD3^+^CD8^+^, CD4^+^/CD8^+^, and CD19^+^ cells in lymphocyte subpopulations between 2 groups (*P* > 0.05), while the percentage of CD16^+^CD56^+^ cells was significantly higher in the MP&ADV group than those in the MP group (*P* < 0.05) (Table 2). In humoral immunity, there was no significant difference in the levels of IgG, IgA, IgM, or IgE (*P* > 0.05, respectively) (Table 2). It was worth mentioning that the levels of IgE in both groups were much higher than the normal range, which is less than 100 kU/L.

### 3.3. *α*-Diversity of the BALF Microbiome

**Figure 1:**
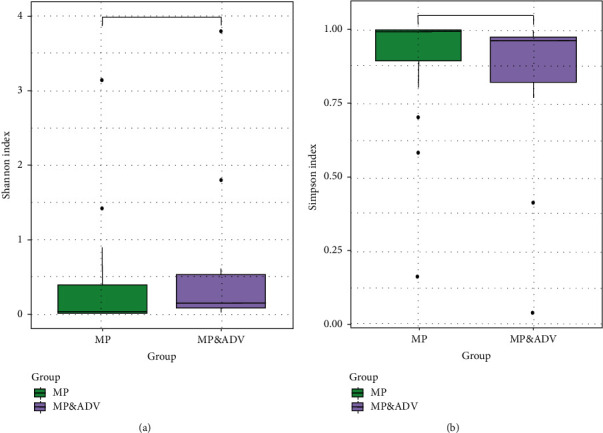
Comparison of the *α*-diversity of microbial communities between the MP and MP&ADV groups. Shannon's index (a) and Simpson index (b) showed no significant difference between the two groups.

The *α*-diversity was determined using the Shannon and Simpson indexes, representing the richness and evenness of the microbiota, respectively. There were no significant differences in the *α*-diversity between the MP and the MP&ADV group (Figure 1).

### 3.4. *β*-Diversity of the BALF Microbiome

**Figure 2:**
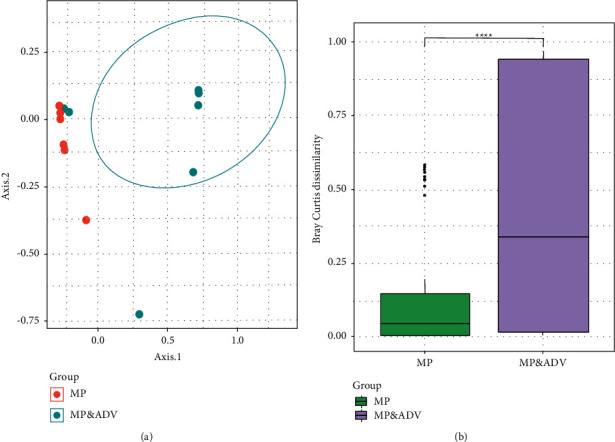
Comparison of the *β*-diversity of microbial communities between the MP and MP&ADV groups. Each plot represents one community from one patient. (a) PCoA plot based on Bray–Curtis distance. Each plot represents one community from one patient. (b) *β*-diversity based on the Bray–Curtis distance of two groups. ^*∗∗∗∗*^*P* < 0.001. PERMANOVA, permutational multivariate analysis of variance.

Principal coordinate analysis (PCoA) provided an overview of the BALF microbiome and reflected the *β*-diversities of the different groups. The *β*-diversity was clearly higher in the MP&ADV group than that in the MP group (Figure 2(a)). In addition, there was a significant difference in the *β*-diversity based on the Bray–Curtis distance between the two groups (*P* < 0.001, PERMANOVA), suggesting that the intragroup difference of the MP group was smaller than that of the MP&ADV group (Figure 2(b)).

### 3.5. The Taxa between the MP and MP&ADV Groups

**Figure 3:**
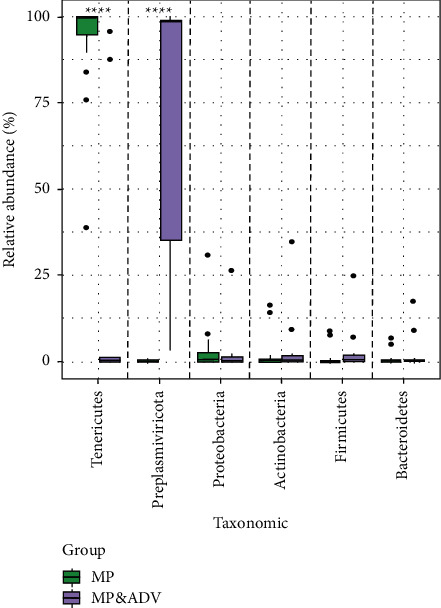
The most abundant phylum (mean relative abundances greater than 0.4% and penetrance greater than 40% among all samples) among the two groups. ^*∗*^*P* < 0.05, ^*∗∗*^*P* < 0.01, and ^*∗∗∗*^*P* < 0.001. Kruskal–Wallis rank-sum test.

The most abundant taxa between the two study groups were compared (Figure 3). The top phylum of the MP BALF microbiota was *Tenericutes* at the phylum level. In contrast, the top phylum of the MP&ADV BALF microbiota was *Preplasmiviricota*. The relative abundance of the two phyla was also significantly different (*P* < 0.001).

### 3.6. Correlation between Microbiota and Clinical Measures

**Figure 4:**
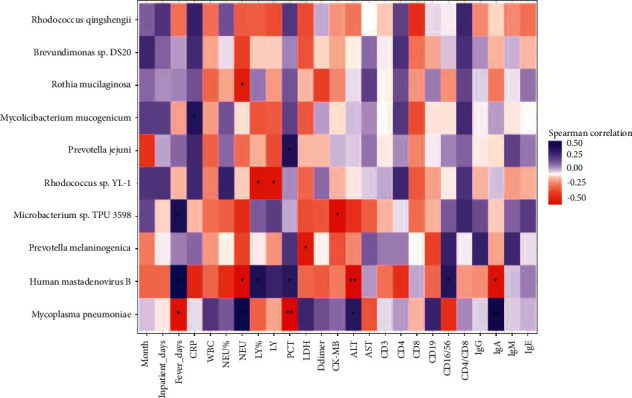
Heatmap of Spearman correlations between clinical measures and microbiota species. WBC, white blood cell counts; LY, lymphocyte count; LY%, lymphocyte percentage; Neu, neutrophil count; Neu%, neutrophil percentage; PCT, procalcitonin; CRP, C-reactive protein; LDH, lactate dehydrogenase; CK-MB, creatine kinase isoenzyme-MB; ALT, alanine aminotransferase; AST, aspartate aminotransferase. ^*∗*^*P* < 0.05 and ^*∗∗*^*P* < 0.01.

Spearman correlation analysis of clinical measures, such as age (months), fever days, inpatient days, CRP, WBC, Neu, Neu%, LY, LY%, PCT, D-dimer, LDH, CK-MB, ALT, AST, and immunological indices, and the most abundant 10 BALF microbiota species was performed. As shown (Figure 4), there was a strong positive correlation (correlation coefficient = 0.53, *P* < 0.01) between *human mastadenovirus B* and fever days, whereas a strong negative correlation (correlation coefficient = -0.50, *P* < 0.01) was observed between *Mycoplasma pneumoniae* and PCT. As far as immunological indices, there was a strong positive correlation (correlation coefficient = 0.45, *P* < 0.01) between *Mycoplasma pneumoniae* and IgA level and a positive correlation (correlation coefficient = 0.36, *P* < 0.05) between *human mastadenovirus B* and percentage of CD16^+^CD56^+^ cells.

## 4. Discussion

In the current study, we found that patients coinfected with *human mastadenovirus B* had a longer duration of fever than those with *M. pneumoniae* monoinfection, and the percentage of CD16^+^CD56^+^ cells was also significantly higher in the coinfection group than those in the MP monoinfection group. In the microbiome study, we found that the BALF microbiome of *M. pneumoniae* monoinfection showed a decline in species richness compared with coinfection; however, the *β*-diversity was higher in the MP&ADV group than that in the MP group. In the correlation between the microbiota and clinical measures, we found that *human mastadenovirus B* was positively correlated with patients' fever days, and *M. pneumonia* was positively correlated with the level of IgA, whereas *M. pneumoniae* was negatively correlated with PCT.

*M. pneumoniae* damages the epithelial cells and cilia of the human airway, promoting mixed infections. The coinfection rate can reach 48% in MPP [9]. HAdV is an important pathogen of respiratory tract infection in children and is responsible for 4–10% of pediatric CAP [11]. Zhou et al. found that HAdV was the most prevalent coinfecting organism in *M. pneumonia* infection and was associated with RMPP [10]. In the current study of RMPP, we found that patients coinfected with *human mastadenovirus B* had a longer fever duration compared with *M. pneumoniae* infection alone, which was consistent with the studies of Zhou et al. and Gao et al. [10, 12], respectively, both of which showed longer fever in the MP&ADV cohort. It seems reasonable that coinfection with HAdV prolongs the clearance time of the pathogen and affects the host immune response, leading to longer inflammation times. In terms of hospital days, we did not find any significant difference between the coinfection and the monoinfection groups. However, Gao's study showed longer hospital stays in coinfection with HAdV [12]. The apparent contradiction may be related to the study populations, which in Gao's study was just MPP patients, whereas they were RMPP patients in ours.

In this study, we found that incidence of *M. pneumoniae*-related extrapulmonary manifestations was around 50% in both groups, and were mainly liver damage and myocardial damage. The incidence was higher than that in the reported data [18–20], which may be associated with the study's populations. As far as the immunological workup, there was no significant difference in the levels of IgG, IgA, IgM, or IgE between the two groups. Some studies reported that serum IgE or atopy was associated with *M. pneumoniae*-related extrapulmonary manifestations [21, 22]. Another study showed that IgE was an independent risk factor for severe adenovirus pneumonia in children [23]. The results are not contradictory because the study populations are different. RMPP patients were our study populations, and the IgE level in our study was higher than that in the reported data [21, 22]. Interestingly, Spearman correlation analysis showed that *M. pneumoniae* was positively correlated with the IgA level, suggesting that *M. pneumoniae* infection is associated with airway mucosal immunity. In addition, we found that the percentage of CD16^+^CD56^+^ cells was significantly higher in the MP&ADV group than that in the MP group. Spearman correlation analysis also showed that *human mastadenovirus B* positively correlated with the percentage of CD16+CD56+ cells. CD16^+^CD56^+^ cells are known as natural killer (NK) cells in humans. NK cells are important mediators of antiviral innate immunity that can be activated by HAdV infection [24, 25].

In the current study, we recruited children of RMPP co-infected with HAdV to study the characteristics of the BALF microbiome composition in these patients in China. The BALF microbiome of *M. pneumoniae* monoinfection showed a decline in species richness compared with coinfection, but the difference was not significant. As regards the *β*-diversity, it was clearly higher in the MP&ADV group than in the MP group in our study, suggesting that the intragroup difference of the MP group was smaller than that of the MP&ADV group. In a study by Wang et al., the bacterial diversity in *M. pneumoniae* pneumonia was found to be lower than that in adenovirus pneumonia [26]. In terms of coinfection with bacteria, *M. pneumoniae* was found to compete for nutrients to eliminate other bacteria [27] and activate the host inflammatory response [28]. As a result, the bacterial diversity was small in MPP [29–31]. In terms of coinfection with the virus, virus infections impair the lung epithelial layer and suppress the immune response, which promotes bacterial outgrowth and frequently leads to secondary bacterial infections [32], and this may increase the bacterial diversity. Consistent with this theory, we also found that when coinfected with HAdV, the bacterial diversity was increased.

Lastly, there were significant relationships between clinical measures and the microbiota, as shown by Spearman correlation analysis. *Human mastadenovirus B* was positively correlated with patients' fever days, indicating that *human mastadenovirus B* may contribute to symptoms. In addition, *M. pneumoniae* was negatively correlated with PCT. *M. pneumoniae* is a bacterial pathogen, and PCT is considered as a biomarker of infections. Our results suggest that the correlations of etiological diagnosis and biomarkers of infection remain unsolved problems.

We would like to mention several potential limitations of this study. First, the sample size was limited, especially in the MP&ADV group. Second, our quantification of the bacteria was only at the DNA level, which cannot distinguish live and dead microorganisms and cannot detect RNA viral genomes. Third, the study population in this study was RMPP patients, which is very limited.

In conclusion, this study showed that the microbiota of the BALF in children with RMPP of *M. pneumoniae* monoinfection was much simpler than those with coinfection with *human mastadenovirus B*. The coinfection was also associated with prolonged fever duration. These results contribute important profiles of the lung microbiota and fill a gap in our knowledge of the mechanism of pathogenesis in RMPP, coinfected with *human mastadenovirus B*.

## Figures and Tables

**Table 1:** Demographical characteristics.

Variable	MP (*n* = 21)	MP&ADV (*n* = 11)	*P* value

Male/Female	11/10	7/4	0.54
Age (months)	71 ± 38	59 ± 18	0.24
Inpatient days (days)	10.38 ± 3.04	10.70 ± 5.25	0.86
Pleural effusion	9 (42.8%)	3 (27.3%)	0.46
Fever days	11.05 ± 3.10	14.56 ± 4.12	0.04
Extrapulmonary manifestation	13 (61.9%)	5 (45.5%)	0.37
Methylprednisolone treatment	21	11	1.0
IVIG treatment	8	7	0.13

**Table 2:** Laboratory findings.

Variable	MP (*n* = 21)	MP&ADV (*n* = 11)	*P* value

WBC counts (×10^9^/L)	12.61 ± 4.98	10.61 ± 6.22	0.34
NEU (%)	72.35 ± 16.06	66.30 ± 20.57	0.09
LY (%)	17.59 ± 9.01	25.28 ± 18.83	0.13
CRP	52.10 ± 48.37	32.13 ± 31.25	0.19
CK-MB	28.81 ± 16.23	25.40 ± 11.82	0.54
PCT	0.26 ± 0.28	0.66 ± 0.72	0.12
LDH (IU/ml)	662.14 ± 338.77	601.82 ± 474.19	0.68
D-dimer (mg/L)	3.25 ± 2.31	2.04 ± 1.07	0.07
ALT (U/L)	54.40 ± 50.92	27.22 ± 44.29	0.14
AST (U/L)	51.03 ± 44.36	52.74 ± 43.58	0.92

Cellular immunity			
CD3^+^T (%)	61.55 ± 15.63	57.66 ± 13.63	0.53
CD3^+^CD4^+^T (%)	34.69 ± 10.70	29.37 ± 7.95	0.20
CD3^+^CD8^+^T (%)	23.77 ± 8.67	24.84 ± 11.56	0.79
CD4^+^/CD8^+^T (%)	1.67 ± 0.64	1.47 ± 0.82	0.50
CD16^+^CD56^+^T (%)	8.54 ± 4.19	15.34 ± 10.67	0.02
CD19^+^T (%)	28.33 ± 14.94	25.30 ± 11.96	0.60

Humoral immunity			
IgG (g/L)	12.22 ± 5.62	11.81 ± 3.08	0.84
IgA (g/L)	1.48 ± 0.51	1.23 ± 0.57	0.25
IgM (g/L)	3.31 ± 2.29	3.45 ± 2.06	0.88
IgE (kU/L)	496.91 ± 809.76	383.34 ± 325.92	0.61

## Data Availability

All the data included in this study are available upon request to the corresponding author.
